# Is orthostatic hypotension and co-existing supine and seated hypertension associated with future falls in community-dwelling older adults? Results from The Irish Longitudinal Study on Ageing (TILDA)

**DOI:** 10.1371/journal.pone.0252212

**Published:** 2021-05-27

**Authors:** Orna A. Donoghue, Matthew D. L. O’Connell, Robert Bourke, Rose Anne Kenny

**Affiliations:** 1 The Irish Longitudinal Study on Ageing (TILDA), Trinity College Dublin, Dublin, Ireland; 2 Department of Population Health Sciences, School of Population Health and Environmental Sciences, King’s College London, London, United Kingdom; 3 Mercer’s Institute for Successful Ageing (MISA), St James’s Hospital, Dublin, Ireland; Universita degli Studi di Napoli Federico II, ITALY

## Abstract

Orthostatic hypotension (OH) often co-exists with hypertension. As increasing age affects baroreflex sensitivity, it loses its ability to reduce blood pressure when lying down. Therefore, supine hypertension may be an important indicator of baroreflex function. This study examines (i) the association between OH and future falls in community-dwelling older adults and (ii) if these associations persist in those with co-existing OH and baseline hypertension, measured supine and seated. Data from 1500 community-dwelling adults aged ≥65 years from The Irish Longitudinal Study on Ageing (TILDA) were used. Continuous beat-to-beat blood pressure was measured using digital photoplethysmography during an active stand procedure with OH defined as a drop in systolic blood pressure (SBP) ≥20 mmHg and/or ≥10 mm Hg in diastolic blood pressure (DBP) within 3 minutes of standing. OH at 40 seconds (OH40) was used as a marker of impaired early stabilisation and OH sustained over the second minute (sustained OH) was used to indicate a more persistent deficit, similar to traditional OH definitions. Seated and supine hypertension were defined as SBP ≥140 mm Hg or DBP ≥90 mm Hg. Modified Poisson models were used to estimate relative risk of falls (recurrent, injurious, unexplained) and syncope occurring over four year follow-up. OH40 was independently associated with recurrent (RR = 1.30, 95% CI = 1.02,1.65), injurious (RR = 1.43, 95% CI = 1.13,1.79) and unexplained falls (RR = 1.55, 95% CI = 1.13,2.13). Sustained OH was associated with injurious (RR = 1.55, 95% CI = 1.18,2.05) and unexplained falls (RR = 1.63, 95% CI = 1.06,2.50). OH and co-existing hypertension was associated with all falls outcomes but effect sizes were consistently larger with seated versus supine hypertension. OH, particularly when co-existing with hypertension, was independently associated with increased risk of future falls. Stronger effect sizes were observed with seated versus supine hypertension. This supports previous findings and highlights the importance of assessing orthostatic blood pressure behaviour in older adults at risk of falls and with hypertension. Observed associations may reflect underlying comorbidities, reduced cerebral perfusion or presence of white matter hyperintensities.

## Introduction

In healthy adults, blood pressure can be maintained within the normal range throughout a range of positions. If the cardiovascular system and neural reflexes are unable to maintain blood pressure on standing from a seated or supine position, the resulting abnormal drop in blood pressure is called orthostatic hypotension (OH). The consensus definition for OH is a sustained drop in blood pressure of ≥20 mmHg in systolic blood pressure (SBP) or ≥10 mm Hg in diastolic blood pressure (DBP) within 3 minutes of standing up [1]. It can be asymptomatic or symptomatic with the typical symptoms including postural dizziness and syncope. OH is often reflective of early autonomic nervous system dysfunction such as disturbed sensitivity of the baroreflex [2].

Not surprisingly, OH is common in older adults, but the prevalence varies widely depending on the population and setting examined. Prevalence in community-dwelling older adults has been reported at 6% [1] while 18.2% of Cardiovascular Health Study participants aged 65 year and over had the condition [3]. There is an increasing prevalence with increasing age [4], increasing frailty status [5] and in those with underlying cardiovascular, renal, neurodegenerative and metabolic conditions [2, 6, 7]. Furthermore, OH independently predicts increased risk of falls [8], increased incident cardiovascular and cerebrovascular disease and all-cause mortality [9] and increased risk of disability, hospitalisation and mortality in those with the highest level of frailty [5]. OH is also associated with syncope, particularly in those with advancing age and increasing comorbidities [10].

Previously, Finucane et al [11] reported associations between OH diagnosed using a beat-to-beat active stand protocol and future falls, injurious falls and unexplained falls in community-dwelling adults aged 50 years and over in The Irish Longitudinal Study on Ageing (TILDA). As Peeters et al [12] reported that middle-aged participants have different risk factor profiles to older adults, this analysis is now being replicated in the ≥65 years age group only and extended to a longer follow-up over four years.

Research has also shown significant heterogeneity in fall risk factors even in older adults, therefore it is important to understand how risk factors may interact or co-exist with each other. One of the most common comorbidities associated with OH is hypertension [13, 14]. This is often linked to severe autonomic dysfunction and reduced ability of the baroreflex to reduce blood pressure when lying down. Fall risk has been reported to increase when OH co-exists with both seated hypertension [11] and supine hypertension [15]. However, it is unclear if it is important to assess for hypertension in both supine and seated positions and/or if a stronger association with falls might be observed when OH co-exists with one over the other.

Therefore, the aims of this study are firstly, to examine the association between OH and future occurrence of recurrent, injurious and unexplained falls and syncope over four year follow-up in community-dwelling adults aged 65 years and older and secondly, to examine these associations in those with co-existing OH and baseline hypertension, measured in both supine and seated positions.

## Materials and methods

### Study design

The Irish Longitudinal Study on Ageing (TILDA) is a prospective cohort study examining the social, economic and health circumstances of community-dwelling adults aged 50 years and older in Ireland. Details of study design and data collection have been described elsewhere [16]. Briefly, the sampling frame is the Irish Geodirectory, a listing of all residential addresses in the Republic of Ireland. A clustered sample of addresses was randomly selected and all household residents aged ≥50 years and their spouses/partners (of any age) were invited to participate at baseline. Ethical approval was obtained from the Faculty of Health Sciences Research Ethics Committee at Trinity College Dublin and all participants provided written informed consent.

### Data collection

Baseline (Wave 1) data from 8,174 participants aged ≥50 years were collected from October 2009 to July 2011; follow-up data were obtained at Wave 2 (February 2012 to March 2013) and Wave 3 (January 2014 to December 2015). Data collection involved three components, (i) a computer-assisted personal interview (CAPI) that included detailed questions on socio-demographics, wealth, health, lifestyle, social support and participation, use of health and social care and attitudes to ageing, conducted by trained social interviewers in the participant’s home, (ii) a self-completion questionnaire (SCQ) completed by participants and returned to the study centre, and (iii) a comprehensive health assessment carried out by research nurses in a dedicated health assessment centre or a modified version carried out in the participant’s home. All three components were included at baseline assessment with follow-up data obtained from the CAPI at Waves 2 and 3.

Inclusion criteria for this analysis was age ≥65 years; Mini-Mental State Examination (MMSE) score ≥18 and no self-reported doctor diagnosis of Parkinson’s disease or cognitive impairment at all wave; availability of valid orthostatic stand data at the baseline health assessment; and available data on falls outcomes obtained from a self or proxy interview at either Wave 2 and/or 3.

### Falls and syncope outcome variables

A number of dichotomous falls variables were derived from data obtained at follow-up (Wave 2 and/or Wave 3). Recurrent falls were defined as two or more falls occurring in the past year or since the last interview. Injurious falls were those in which a participant reported that they had injured themselves seriously enough to require medical attention. Unexplained falls were those in which a participant could not identify the reason for the fall. Syncope was defined as a faint or blackout occuring in the past year or since the last interview.

### Cardiovascular function

Average seated blood pressure was measured using an Omron blood pressure monitor (two readings 1 minute apart). Hypertension was defined as SBP ≥140 mm Hg or DBP ≥90 mm Hg [17]. Non-invasive continuous beat-to-beat blood pressure was measured during an active stand procedure using digital photoplethysmography (Finometer MIDI Device, Finapres Medical Systems BV, Amsterdam, The Netherlands) as outlined previously [4]. Participants rested supine for 10 minutes and were then asked to quickly stand; a nurse assisted if necessary. SBP, DBP and heart rate were recorded during the 10 minutes rest period and for 2 minutes after standing. Supine hypertension was defined as SBP of ≥140 mm Hg or DBP of ≥90 mm Hg [17] using the mean blood pressure data during the first 30 seconds of the minute before standing. OH was defined as a drop in blood pressure of ≥20 mmHg in SBP and/or ≥10 mm Hg in DBP within 3 minutes of standing up [1]. Two variants of OH were used for analysis. Impaired stabilisation (OH40) was defined as a drop in SBP of ≥20 mm Hg or DBP of ≥10 mm Hg at 40 seconds [11]. Sustained OH was defined as a drop in SBP of ≥20 mm Hg or DBP of ≥10 mm Hg sustained at all timepoints between 60 and 120 seconds after standing. In addition, the SBP and DBP drop (difference from baseline) at 40 seconds and 120 seconds were recorded [18]. Self-reported postural dizziness during the active stand was also recorded.

### Other covariates obtained at baseline

Socio-demographic variables such as age, sex, education (primary, secondary and tertiary education corresponding to ≤8, 9–13 and >13 years) and living status (living alone or with others) were obtained. Participants self-reported the following doctor-diagnosed cardiovascular conditions (angina, heart attack, stroke or mini-stroke, diabetes, heart murmur and abnormal heart rhythm) and non-cardiovascular conditions (osteoporosis, arthritis, cataracts, glaucoma, age-related macular degeneration, cancer). Participants were also asked if they had ever had a hip or wrist fracture. Participants reported all medications taken regularly and the following medications were identified using Anatomical Therapeutic Chemical (ATC) Classification codes: alpha blockers (ATC codes: C02CA, C02LE), beta blockers (ATC code: C07), calcium channel blockers (ATC code: C08), diuretics (ATC code: C03), angiotensin-converting enzyme (ACE) inhibitors (ATC code: C09) and antidepressants (ATC codes: NO6A). Participants reported a history of falls in the past year (categorised as 0, 1 or ≥2 falls). Depressive symptoms were assessed using the 20-item Centre for Epidemiological Studies Depression (CES-D) scale [19].

During the health assessment, height and weight were measured using a 240 wall-mounted measuring rod and electronic floor scales respectively (SECA, Birmingham, UK), allowing body mass index (BMI) to be calculated. Mean grip strength was obtained from two trials on each hand using a hydraulic hand dynamometer (Baseline^®^, Fabrication Enterprises, Inc., White Plains, NY). Gait speed was measured with a computerised walkway (active area 4.88 m) with embedded pressure sensors (GAITRite^®^, CIR Systems Inc, New York, USA). Participants walked for 2.5 m before and 2 m after the mat to allow for acceleration and deceleration. The mean of two trials was calculated. Global cognition was assessed using the MMSE [20] and Montreal Cognitive Assessment (MoCA) [21].

### Statistical analysis

Baseline characteristics of the full sample were summarised. Modified Poisson models were used to estimate relative risk of falls (recurrent, injurious, unexplained) or syncope occurring over approximately four years follow-up. For each outcome, Model 1 included a limited number of socio-demographic covariates (age, sex, education, living status), the number of follow-up waves available (1 or 2) and follow-up time. Model 2 included the variables included in Model 1 plus angina, heart attack, diabetes, stroke or transient ischaemic attack (TIA), heart murmur, irregular heart rhythm, cataracts, glaucoma, age-related macular degeneration, cancer, arthritis, MOCA, CES-D, postural dizziness, baseline SBP and heart rate, gait speed, grip strength, BMI, alpha blockers, beta blockers, calcium channel blockers, diuretics, ACE inhibitors and antidepressants. The analysis was then repeated after stratifying by presence or absence of (i) supine hypertension and (ii) seated hypertension. The main analysis did not adjust for falls history as, while this is a strong risk factor for future falls, it was considered non-causative and possibly related to pre-existing OH or other included risk factors prior to baseline. A sensitivity analysis additionally adjusting for history of falls at baseline and history of hip or wrist fracture is included as a supplement. In addition, correlations were obtained to examine the relationship between SBP and DBP in both seated and supine positions and also between supine blood pressure and blood pressure deficits at 40 seconds and 120 seconds after standing. The number of participants with hypertension was calculated and compared when using supine and seated blood pressure. Statistical analysis was conducted using Stata v14.1 (StataCorp LP, Texas, USA).

## Results

**Fig 1:**
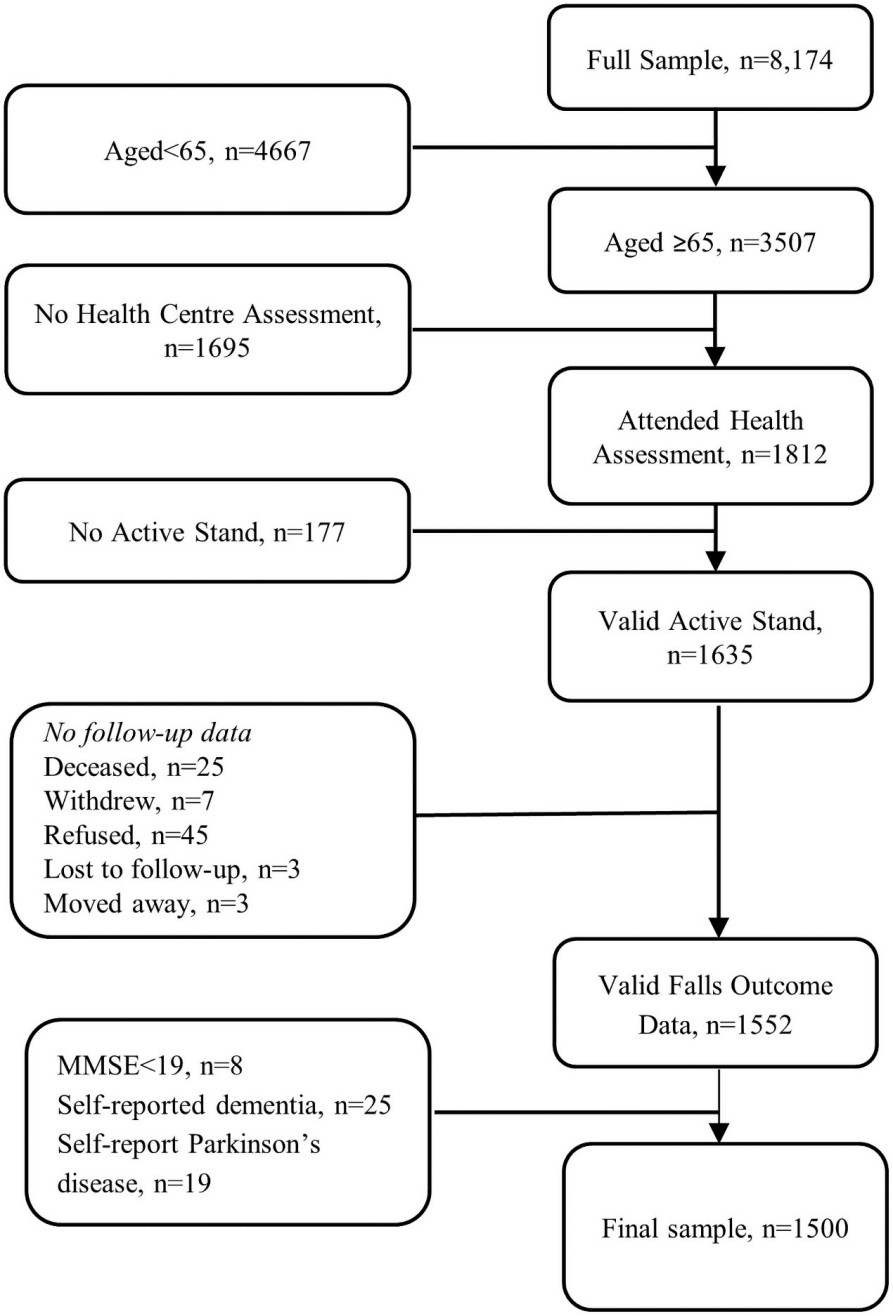
Flowchart of participants included in this analysis.

After exclusion criteria were applied, there were 1500 eligible participants for this analysis (see flowchart in Fig 1). In this population-based sample (mean age 71.1 years, range 65–91 years; 51.0% female), 307 participants (20.5%) reported recurrent falls, 305 (20.3%) reported injurious falls, 184 (12.3%) reported unexplained falls and 74 (4.9%) reported syncope at either wave 2 or 3 i.e. over 4 year follow-up. Baseline characteristics of the sample are provided in Table 1. Mean follow-up time was 4.1 years (range 2.8–5.7 years). 1326 (88.4%) participants had falls or syncope data at both wave 2 and wave 3, 138 (9.2%) at wave 2 only and 36 (2.4%) at wave 3 only.

**Table 1:** Sample description (based on participants with active stand data available at baseline and recurrent falls data available at follow-up).

Age (years, mean (SD))	71.1 (5.2)
Female (count (%))	765 (51.0)
Education	
Primary (count (%))	458 (30.6)
Secondary (count (%))	546 (36.4)
Higher (count (%))	495 (33.0)
Household	
Living alone (count(%))	371 (24.7)
Living with others (count(%))	1129 (75.3)
Angina (count(%))	130 (8.7)
Heart attack (count(%))	105 (7.0)
Diabetes (count(%))	135 (9.0)
Stroke or tia (count(%))	76 (5.1)
Heart murmur (count(%))	96 (6.4)
Abnormal heart rhythm (count(%))	162 (10.8)
Cataracts (count(%))	254 (17.0)
Glaucoma (count(%))	49 (3.3)
ARMD (count(%))	47 (3.1)
Cancer (count(%))	117 (7.8)
Arthritis (count(%))	564 (37.6)
Falls in last year	
1 (count (%))	232 (15.5)
2+ (count (%))	111 (7.4)
Hx of hip or wrist fracture (count (%))	199 (13.5)
Alpha blockers (count (%))	40 (2.7)
Beta blockers (count (%))	300 (20.0)
Calcium channel blockers (count (%))	218 (14.5)
Diuretics (count (%))	161 (10.7)
ACE inhibitors (count (%))	498 (33.2)
Antidepressants (count (%))	83 (5.5)
Seated systolic BP	140.0 (20.1)
Seated diastolic BP	81.0 (11.3)
Supine systolic BP	140.7 (24.1)
Supine diastolic BP	72.0 (11.6)
OH40	314 (20.9)
Sustained OH	123 (8.2)
Dizziness on standing	551 (36.7)
Recurrent falls at waves 2 & 3	307 (20.5)
Injurious falls at waves 2 & 3	305 (20.3)
Unexplained falls at waves 2 & 3	184 (12.3)
Syncope at waves 2 & 3	149 (10.0)

ACE, angiotensin-converting enzyme; BP, blood pressure; OH40, orthostatic hypotension at 40 seconds; TIA, transient ischaemic attack.

All falls outcomes occurred more frequently in participants with either OH40 or sustained OH than those without (Table 2). After adjustment for demographic characteristics in the regression analysis, OH40 was associated with recurrent falls (RR = 1.35, 95% 1CI = 1.08,1.68); injurious falls (RR = 1.44, 95% CI = 1.16,1.78) and unexplained falls (RR = 1.57, 95% CI = 1.16,2.13). Adjustment for health-related covariates did not greatly affect these relationships (Table 3). Sustained OH was also associated with injurious (RR = 1.55, 95% CI = 1.18,2.05) and unexplained falls (RR = 1.63, 95% CI = 1.06,2.50), but not recurrent falls, after adjustment for demographic and health-related covariates.

**Table 2:** Incidence of falls outcomes at follow-up by OH categories (OH40 and sustained OH) and presence or absence of hypertension measured in both supine and seated positions.

	Recurrent falls	Injurious falls	Unexplained falls	Syncope
N	1498	1500	1500	1494
Total	307 (20.5)	305 (20.3)	184 (12.3)	149 (10.0)
OH40				
OH40 -ve (79.1%)	224 (18.9)	216 (18.2)	128 (10.8)	110 (9.3)
OH40 +ve (20.9%)	83 (26.5)	89 (28.3)	56 (17.8)	39 (12.5)
Sustained OH				
OHsus -ve (91.8%)	276 (20.1)	264 (19.2)	161 (11.7)	131 (9.6)
OHsus +ve (8.2%)	31 (25.2)	41 (33.3)	23 (18.7)	18 (14.6)
Using supine blood pressure (n = 1500)				
Normotensive (n = 757)	158 (20.9)	151 (20.0)	92 (12.2)	74 (9.8)
Hypertensive (n = 743)	149 (20.1)	154 (20.7)	92 (12.4)	75 (10.1)
OH40				
OH40 & HTN- (17.6%)	35 (26.3)	34 (25.6)	25 (18.8)	20 (15.2)
OH40 & HTN+ (24.4%)	48 (26.7)	55 (30.4)	31 (17.1)	19 (10.6)
Sustained OH				
OHsus & HTN- (5.9%)	14 (31.1)	17 (37.8)	9 (20.0)	8 (17.8)
OHsus & HTN+ (10.5%)	17 (21.8)	24 (30.8)	14 (18.0)	10 (12.8)
Using seated blood pressure (n = 1491)				
Normotensive (n = 764)	154 (20.2)	161 (21.1)	93 (12.2)	75 (9.9)
Hypertensive (n = 727)	152 (21.0)	144 (19.8)	90 (12.4)	73 (10.1)
OH40				
OH40 & HTN- (19.0%)	32 (22.1)	33 (22.8)	20 (13.8)	19 (13.2)
OH40 & HTN+ (23.0%)	51 (30.7)	56 (33.5)	36 (21.6)	20 (12.1)
Sustained OH				
OHsus & HTN- (7.3%)	12 (21.4)	16 (28.6)	7 (12.5)	8 (14.3)
OHsus & HTN+ (9.2%)	19 (28.4)	25 (37.3)	16 (23.9)	10 (14.9)

ACE, angiotensin-converting enzyme; HTN-, not classified as having hypertension; HTN+, classified as having hypertension; OH40, orthostatic hypotension at 40 seconds. Note that recurrent falls data were available for 1498 participants, injurious and unexplained falls data for 1500 participants and syncope data for 1494 participants.

**Table 3:** Relative risk of falls outcomes at follow-up by OH categories (OH40 and sustained OH).

	Model 1 RR (95%CI)	Model 2 RR (95%CI)
Recurrent falls		
OH40	1.35 [1.08,1.68]**	1.30 [1.02,1.65]*
Sustained OH	1.19 [0.87,1.63]	1.26 [0.90,1.76]
n	1497	1433
Injurious falls		
OH40	1.44 [1.16,1.78]***	1.43 [1.13,1.79]**
Sustained OH	1.52 [1.17,1.98]**	1.55 [1.18,2.05]**
n	1499	1434
Unexplained falls		
OH40	1.57 [1.16,2.13]**	1.55 [1.13,2.13]**
Sustained OH	1.49 [1.00,2.23]*	1.63 [1.06,2.50]*
n	1499	1434
Syncope		
OH40	1.19 [0.84,1.68]	1.01 [0.69,1.47]
Sustained OH	1.45 [0.94,2.24]	1.04 [0.65,1.67]
n	1493	1428

ACE, angiotensin-converting enzyme; OH40, orthostatic hypotension at 40 seconds.

Model 1: Age, sex, education, living alone or with others, follow up time & number of follow up waves available.

Model 2: Model 1 + angina, heart attack, diabetes, stroke or TIA, heart murmur, irregular heart rhythm, cataracts, glaucoma, age-related macular degeneration, cancer, arthritis, MOCA, CES-D, postural dizziness, baseline systolic blood pressure and heart rate, gait speed, grip strength, BMI, alpha blockers, beta blockers, calcium channel blockers, diuretics, ACE inhibitors & antidepressants.

*p<0.05,

**p<0.01,

***p<0.001.

**Fig 2:**
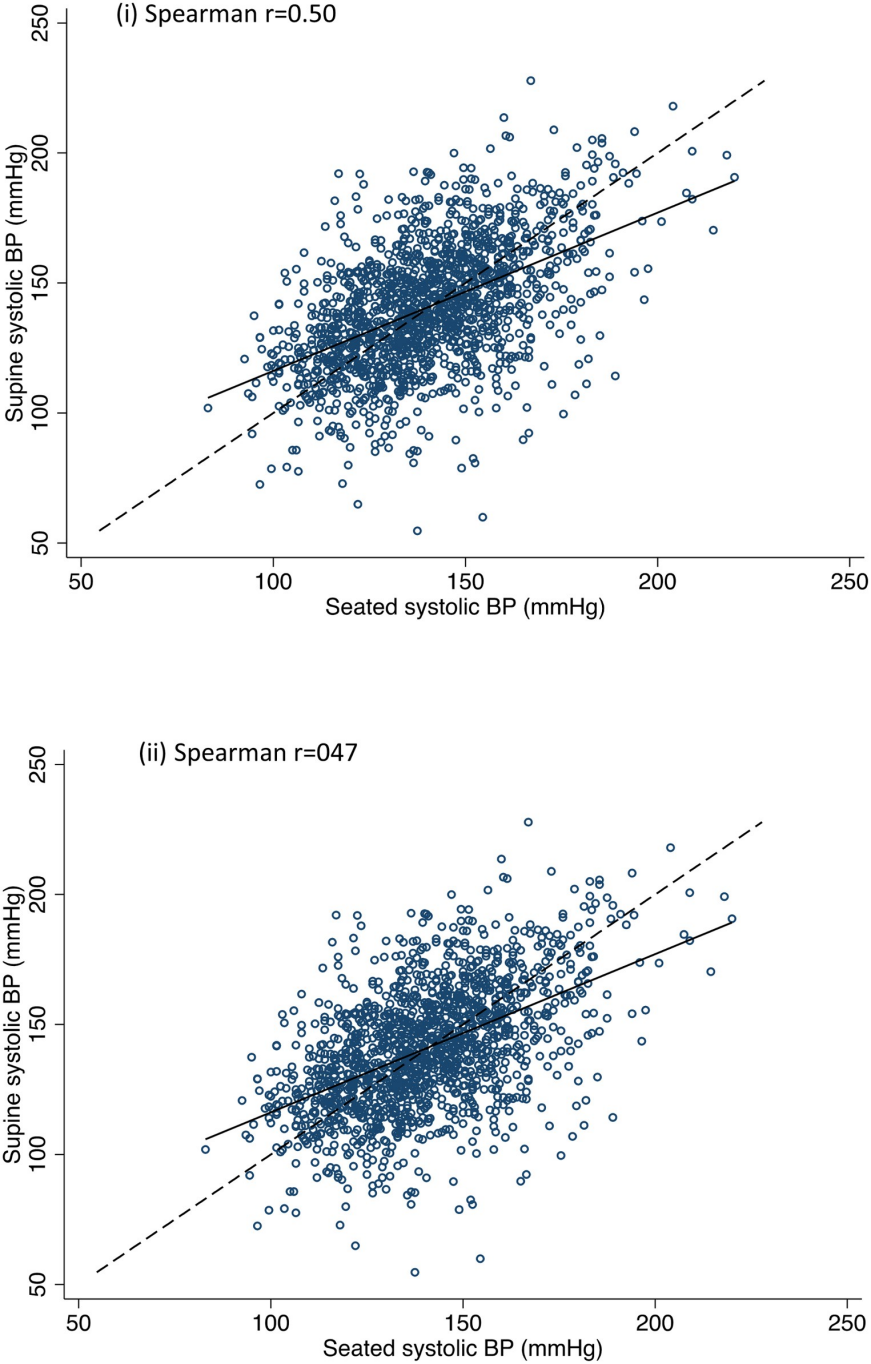
Relationship between (i) seated and supine SBP and (ii) seated and supine DBP.

Fig 2 shows the relationship between SBP and DBP measured in the seated and supine positions. Measurements were similarly correlated in both positions (r = 0.47–0.50). SBP was centred around a similar mean in both positions, while DBP measurements tended to be higher in the seated position (see Table 1). Using supine blood pressure, 757 participants were classified as normotensive while 743 had hypertension. Using seated blood pressure, 764 participants had normal blood pressure while 727 had hypertension. Blood pressure measured in the seated and supine positions agreed on the classification of 68.3% of normotensives and 68.1% of hypertensives.

Correlations between supine blood pressure and blood pressure deficits at 40 seconds and 120 seconds after standing were low (SBP: r = -0.13 and r = -0.15 respectively; DBP: r = -0.11 and r = -0.15 respectively). Correlations were even lower for seated blood pressure and deficits in SBP and DBP at 40 seconds and 120 seconds (r = 0.01–0.03 for all).

Table 2 shows the frequency of the different fall outcomes and syncope across the different OH (OH40, sustained OH) and blood pressure (normotensive, hypertensive) classifications. Frequency of all falls outcomes were higher in those with OH40 or sustained OH compared to those without. When stratified by hypertension categories, frequency of falls outcomes were higher in participants with co-existing OH and seated hypertension compared to normotension, with more variable results for supine measures. Levels of antihypertensive use were generally similar in participants with and without physiological hypertension, except for higher use of ACE inhibitors (37.0% vs 29.3%) in supine normotensives compared to supine hypertensives (S1 Table).

When the regression analysis was repeated to examine the effect of the presence or absence of hypertension, there were observed associations of at least borderline statistical significance between OH40 and co-existing seated and supine hypertension for all falls outcomes (Table 4). There were also associations between sustained OH and co-existing seated and supine hypertension with injurious and unexplained falls (Table 4). The magnitude of these effects was consistently larger when hypertension was measured in the seated compared to the supine position. Sustained OH co-existing with supine and seated normotension was also associated with injurious falls, while OH40 and supine normotension was associated with unexplained falls. There were no clear associations with syncope for any combination of OH and hypertension measurements.

**Table 4:** Relative risk of falls outcomes at follow-up by OH categories (OH40 and sustained OH) stratified by supine or seated hypertension.

	Recurrent falls RR (95%CI)	Injurious falls RR (95%CI)	Unexplained falls RR (95%CI)	Syncope RR (95%CI)
OH40				
HTN- (supine)	1.26 [0.86,1.84]	1.30 [0.90,1.89]	1.72 [1.07,2.77]*	1.09 [0.59,2.04]
HTN+ (supine)	1.52 [1.10,2.09]*	1.55 [1.14,2.10]**	1.54 [1.00,2.39]	0.91 [0.53,1.55]
Sustained OH				
HTN- (supine)	1.39 [0.84,2.30]	1.66 [1.08,2.55]*	1.53 [0.74,3.18]	0.92 [0.37,2.26]
HTN+ (supine)	1.45 [0.90,2.34]	1.49 [1.00,2.22]*	2.05 [1.16,3.62]*	0.91 [0.48,1.75]
OH40				
HTN- (seated)	1.04 [0.70,1.54]	1.11 [0.78,1.58]	1.31 [0.77,2.23]	1.19 [0.71,2.01]
HTN+ (seated)	1.59 [1.16,2.17]**	1.87 [1.38,2.54]***	2.14 [1.42,3.24]***	0.81 [0.46,1.42]
Sustained OH				
HTN- (seated)	1.09 [0.64,1.85]	1.46 [0.94,2.27]	1.27 [0.57,2.81]	1.35 [0.70,2.58]
HTN+ (seated)	1.52 [0.97,2.39]	1.75 [1.19,2.58]**	2.51 [1.50,4.21]***	0.79 [0.42,1.49]

ACE, angiotensin-converting enzyme; HTN-, not classified as having hypertension; HTN+, classified as having hypertension; OH40, orthostatic hypotension at 40 seconds.

Adjusted for age, sex, education, living alone or with others, follow-up time, number of follow-up waves available, angina, heart attack, diabetes, stroke or TIA, heart murmur, irregular heart rhythm, cataracts, glaucoma, age-related macular degeneration, cancer, arthritis, MOCA, CES-D, postural dizziness, baseline systolic blood pressure and heart rate, gait speed, grip strength, BMI, alpha blockers, beta blockers, calcium channel blockers, diuretics, ACE inhibitors, antidepressants.

*p<0.05,

**p<0.01,

***p<0.001.

Sensitivity analyses showed that adjusting for history of falls and fractures mildly reduced the strength of the OH and falls relationships (S2 Table). Effects remained statistically significant with the exception of the associations between co-existing OH40 and supine hypertension with unexplained falls, and between sustained OH and coexisting supine hypertension and injurious falls.

## Discussion

In this analysis, we found that OH at baseline (OH40 and sustained OH) was independently associated with an increased risk of future recurrent, injurious and unexplained falls in community-dwelling adults aged 65 years and older. OH40 co-existing with hypertension, measured both seated and supine, was associated with increased risk of all outcomes, however stronger effect sizes were observed with seated versus supine hypertension. There were also associations observed between OH40 with co-existing normotension and injurious and unexplained falls. As we controlled for antihypertensives, it is possible that these normotensives have controlled hypertension but may still have other pre-existing risk factors for hypertension such as higher age, smoking and kidney disease, all of which have also been associated with OH in a recent SPRINT trial [22]. This may partly explain the observed associations with falls in those with OH but normal blood pressure.

In a systematic review and meta-analysis, Mol et al [8] concluded that OH is associated with falls in older adults, independent of study population, study design and quality, OH definition and blood pressure measurement method. The inclusion of longitudinal studies in this review suggested that OH may be a cause rather than a consequence of falls. The results of this analysis are consistent with these previous findings and highlight the importance of assessing orthostatic blood pressure behaviour in older adults at risk of falls and who are hypertensive.

OH may be directly linked to falls due to reduced cerebral perfusion and a subsequent reduction in oxygenation in the brain within seconds of standing up from a lying or seated position [23]. OH has also been associated with cerebral white matter hyperintensities [24], which have been associated with deficits in gait [25], cognitive function [26] and mood [27], all of which may indirectly link OH and falls. The prevalence of hypertension increases with age and is also associated with white matter lesions [28], therefore it is not surprising that there is a strong association between OH and hypertension [15].

The clinical syndrome of supine hypertension and OH is well recognised and often challenging to treat clinically [29]. In this study, OH and co-existing hypertension was associated with an increased risk of falls, regardless of whether hypertension was measured supine or seated although associations were stronger when seated. Although there are some inconsistencies in the literature, seated blood pressure has been reported to be higher compared to supine values [30, 31]. However, this may be due to a sequencing effect, as this finding is often reported in studies that measure seated blood pressure first (as in this study); with the opposite also holding true [31].

The clearer stratification of OH effects with seated BP in this analysis may also be due to more accurate resting measurements from oscillometric compared to finometer blood pressure, the 140/90 mmHg cut-point being more suitable for seated blood pressure or from the greater contribution of the higher seated diastolic measurements to the hypertension category. While 140/90 mm Hg was used in this study, there is no formal definition for supine hypertension. Krzesinski et al [32] reported that a supine blood pressure cut-off of ≥130/80 mmHg was a sensitive and specific predictor of hypertension detected using ambulatory blood pressure monitoring while other authors have proposed a cut-off of ≥150/90 mmHg [33]. From a clinical perspective however, it appears that seated blood pressure is as useful, if not more useful than supine blood pressure when examining falls risk in older adults with OH.

Levels of antihypertensive use were similar in participants with and without hypertension, in line with an earlier analysis showing 50% treatment efficacy in the TILDA population [34] and correlations between resting blood pressure and size of the blood pressure drop were low. This suggests that the generally stronger effects observed in those with hypertension are not explained by medication use or by larger drops in blood pressure on standing, however it is possible that they reflect a difference in cerebral autoregulation.

### Implications

The American Geriatrics Society/British Geriatrics Society guidelines [35] for falls prevention in community-dwelling adults include cardiovascular assessment and management of postural hypotension within a multifactorial intervention. The most common causes of postural hypotension include dehydration, medications, and autonomic neuropathy, therefore not surprisingly, successful multifactorial fall prevention programs have typically included hydration, medication reviews to remove unnecessary medications and prescription of specific medications (e.g., fludrocortisone and midodrine) and/or other strategies to reduce postural hypotension (e.g. elastic stockings, abdominal binders) [35]. Currently, there are no clinical trials examining the effect of treatment of OH on falls, however the results of randomised clinical trials and cohort studies do suggest that cardiovascular evaluation and interventions may reduce falls in patient populations [36].

The current results also support evaluating OH in older adults with hypertension, especially if their BP is poorly controlled. There is sometimes reluctance clinically to aggressively treat hypertension in older adults for fear of increasing the risk of OH, falls and syncope, however recent evidence suggests that intensive treatment of hypertension is beneficial, with studies reporting a decreased prevalence of OH [37] and increased cerebral blood flow [38]. In addition, OH was not associated with a higher risk of cardiovascular disease events, falls or syncope when taking blood pressure treatment strategy i.e. standard and aggressive treatment, into account [39]. In contrast, Gangavati et al [15] reported that patients with uncontrolled hypertension and OH were at greater risk of falls than those with controlled hypertension with or without OH, however caution is advised in specific circumstances such as in those with autonomic failure or in those who cannot tolerate lower BP levels [15]. More recently, Benetos et al [40] recommended treatment of hypertension in the oldest old should be done according to function and frailty status. Furthermore, Liguori et al [5] highlighted the importance of including OH assessment within a comprehensive geriatric assessment. This would allow frailty and consequently, the risk of other outcomes including disability, hospitalisation and mortality to be assessed.

### Strengths and limitations

Strengths of this study include the large, nationally representative sample of community-dwelling older adults, the ability to detect OH using beat-to-beat blood pressure monitoring which has been suggested to have the largest clinical relevance [8] and the availability of multiple confounders including self-report and objective measurements across multiple domains.

Fall diaries with regular follow-up was not feasible in this study, therefore one limitation is the use of self-reported falls in the past year/since the last interview. Yearly recall of falls has been found to have a sensitivity of 91–95% and a specificity of 80–89% and while it is subject to recall bias, recurrent falls (i.e. 2 or more falls) and injurious falls should be less susceptible to this [41]. In addition, Frith et al [42] suggested that time to event analysis may be more appropriate than binary approaches like logistic analysis as falls are multifactorial and have complex interactions between risk factors which may change over time. Blood pressure was measured in the supine, seated and standing positions. However, it was not possible to reliably separate postural drops on transition from supine to seated and seated to standing as this was not included in the active standing protocol and seated measurements used a different technique and protocol. Finally, sample sizes for the hypertension stratified analyses were relatively small. Future studies in larger samples are required to more formally assess interactions and precisely estimate size of effects between resting blood pressure, orthostatic drops and falls outcomes.

## Conclusions

In conclusion, OH, particularly when co-existing with hypertension, was independently associated with an increased risk of future falls, but not syncope, in community-dwelling adults aged 65 years and older. Stronger effect sizes were observed when hypertension was measured seated although effects were also observed with supine hypertension. There were also associations observed between OH40 and unexplained falls and between sustained OH and injurious falls in normotensives. The current analysis provides further support to highlight the importance of including a structured cardiovascular assessment within a multifactorial fall prevention assessment to reduce the risk of adverse outcomes associated with falls such as injury, fracture, reduced independence, premature nursing home admission and death [43].

## Supporting information

S1 Table Use of medications by hypertension status. (DOCX)

S2 Table Relative risk of falls and syncope outcomes at follow-up by OH categories (OH40 and sustained OH) stratified by supine or seated hypertension, with additional adjustment for baseline history of falls and fractures. HTN-, not classified as having hypertension; HTN+, classified as having hypertension; OH40, orthostatic hypotension at 40 seconds. Adjusted for age, sex, education, living alone or with others, follow-up time, number of follow-up waves available, angina, heart attack, diabetes, stroke or TIA, heart murmur, irregular heart rhythm, cataracts, glaucoma, age-related macular degeneration, cancer, arthritis, MOCA, CES-D, postural dizziness, baseline systolic blood pressure and heart rate, gait speed, grip strength, BMI, alpha blockers, beta blockers, calcium channel blockers, diuretics, ACE inhibitors, antidepressants, history of falls in last year, history of adult hip or wrist fracture. *p<0.05, **p<0.01, ***p<0.001.
(DOCX)
