# Supplementary material for: Is orthostatic hypotension and co-existing supine and seated hypertension associated with future falls in community-dwelling older adults? Results from The Irish Longitudinal Study on Ageing (TILDA)
Source: PLoS One. 2021 May 27;16(5):e0252212. doi: 10.1371/journal.pone.0252212 (PMC8158994; doi:10.1371/journal.pone.0252212)
Supplement: S1 Table — (DOCX) [file pone.0252212.s001.docx]

**S1 Table. Use of medications by hypertension status.**

|  | Normotensive | Hypertensive |
| --- | --- | --- |
| Supine BP |  |  |
| Alpha blockers, n (%) | 16 (2.1) | 24 (3.2) |
| Beta blockers, n (%) | 165 (21.8) | 135 (18.2) |
| Calcium channel blockers, n (%) | 112 (14.8) | 106 (14.3) |
| Diuretics, n (%) | 73 (9.6) | 88 (11.8) |
| ACE inhibitors, n (%) | 280 (37.0) | 218 (29.3) |
| Antidepressants, n (%) | 41 (5.4) | 42 (5.7) |
| Seated BP |  |  |
| Alpha blockers, n (%) | 16 (2.1) | 23 (3.2) |
| Beta blockers, n (%) | 154 (20.2) | 143 (19.7) |
| Calcium channel blockers, n (%) | 98 (12.8) | 117 (16.1) |
| Diuretics, n (%) | 76 (10.0) | 83 (11.4) |
| ACE inhibitors, n (%) | 256 (33.5) | 238 (32.7) |
| Antidepressants, n (%) | 48 (6.3) | 35 (4.8) |
| ACE, Angiotensin converting enzyme | | |
