# Supplementary material for: Is orthostatic hypotension and co-existing supine and seated hypertension associated with future falls in community-dwelling older adults? Results from The Irish Longitudinal Study on Ageing (TILDA)
Source: PLoS One. 2021 May 27;16(5):e0252212. doi: 10.1371/journal.pone.0252212 (PMC8158994; doi:10.1371/journal.pone.0252212)
Supplement: S2 Table — HTN-, not classified as having hypertension; HTN+, classified as having hypertension; OH40, orthostatic hypotension at 40 seconds. Adjusted for age, sex, education, living alone or with others, follow-up time, number of follow-up waves available, angina, heart attack, diabetes, stroke or TIA, heart murmur, irregular heart rhythm, cataracts, glaucoma, age-related macular degeneration, cancer, arthritis, MOCA, CES-D, postural dizziness, baseline systolic blood pressure and heart rate, gait speed, grip strength, BMI, alpha blockers, beta blockers, calcium channel blockers, diuretics, ACE inhibitors, antidepressants, history of falls in last year, history of adult hip or wrist fracture. *p<0.05, **p<0.01, ***p<0.001. (DOCX) [file pone.0252212.s002.docx]

| **S2 Table. Relative risk of falls and syncope outcomes at follow-up by OH categories (OH40 and sustained OH) stratified by supine or seated hypertension, with additional adjustment for baseline history of falls and fractures** | | | | |
| --- | --- | --- | --- | --- |
|  | Recurrent falls | Injurious falls | Unexplained falls | Syncope |
|  | RR (95%CI) | RR (95%CI) | RR (95%CI) | RR (95%CI) |
| OH40 |  |  |  |  |
| HTN- (supine) | 1.20 [0.83,1.74] | 1.17 [0.80,1.72] | 1.66 [1.04,2.65]* | 1.10 [0.59,2.06] |
| HTN+ (supine) | 1.50 [1.08,2.08]* | 1.46 [1.07,2.00]* | 1.44 [0.91,2.29] | 0.86 [0.49,1.50] |
| Sustained OH |  |  |  |  |
| HTN- (supine) | 1.34 [0.82,2.20] | 1.47 [0.94,2.29] | 1.50 [0.74,3.04] | 0.85 [0.35,2.11] |
| HTN+ (supine) | 1.54 [0.97,2.47] | 1.44 [0.95,2.17] | 2.09 [1.14,3.82]* | 0.86 [0.44,1.69] |
| OH40 |  |  |  |  |
| HTN- (seated) | 1.01 [0.67,1.51] | 0.99 [0.69,1.41] | 1.13 [0.66,1.95] | 1.22 [0.72,2.07] |
| HTN+ (seated) | 1.52 [1.11,2.08]** | 1.77 [1.29,2.42]*** | 1.97 [1.31,2.98]** | 0.66 [0.36,1.21] |
| Sustained OH |  |  |  |  |
| HTN- (seated) | 1.21 [0.71,2.05] | 1.44 [0.92,2.23] | 1.17 [0.51,2.72] | 1.41 [0.74,2.68] |
| HTN+ (seated) | 1.40 [0.90,2.19] | 1.55 [1.03,2.33]* | 2.34 [1.41,3.87]*** | 0.58 [0.30,1.13] |
| HTN-, not classified as having hypertension; HTN+, classified as having hypertension; OH40, orthostatic hypotension at 40 seconds. | | | | |
| Adjusted for age, sex, education, living alone or with others, follow-up time, number of follow-up waves available, angina, heart attack, diabetes, stroke or TIA, heart murmur, irregular heart rhythm, cataracts, glaucoma, age-related macular degeneration, cancer, arthritis, MOCA, CES-D, postural dizziness, baseline systolic blood pressure and heart rate, gait speed, grip strength, BMI, alpha blockers, beta blockers, calcium channel blockers, diuretics, ACE inhibitors, antidepressants, history of falls in last year, history of adult hip or wrist fracture. | | | | |
| *p<0.05, **p<0.01, ***p<0.001 | |  |  |  |
